# Efficacy of Increased Salt and Water Intake on Pediatric Vasovagal Syncope: A Meta-Analysis Based on Global Published Data

**DOI:** 10.3389/fped.2021.663016

**Published:** 2021-05-13

**Authors:** Yaru Wang, Yuanyuan Wang, Xueying Li, Junbao Du, Hao Zhang, Hongfang Jin, Ying Liao

**Affiliations:** ^1^Department of Pediatrics, Peking University First Hospital, Beijing, China; ^2^Department of Medical Statistics, Peking University First Hospital, Beijing, China; ^3^Key Laboratory of Molecular Cardiovascular Sciences, Ministry of Education, Beijing, China; ^4^Department of Cardiothoracic Surgery, Shanghai Children's Medical Center, Shanghai Jiao Tong University School of Medicine, Shanghai, China

**Keywords:** meta-analysis, salt, water, therapeutic efficacy, children, vasovagal syncope

## Abstract

**Objective:** This study was designed to assess the efficacy of increased salt and water intake in the treatment of pediatric vasovagal syncope (VVS) based on a meta-analysis of global data.

**Methods:** Following the established inclusion criteria, seven databases, Cochrane Library, EMBASE, PubMed, Web of Science, VIP, Wanfang, and China National Knowledge Infrastructure (CNKI), were searched using specific terms. The Cochrane Bias risk assessment tool was used as a quality assessment tool of the included studies, and publication bias was assessed by funnel plots. Review Manager 5.4 software was used to analyze the efficacy of the included studies, taking the negative changing rate of the head-up tilt test (HUTT) and recurrence rate of syncope or presyncope as therapeutic efficacy evaluations.

**Results:** In total, 5 randomized controlled trials (RCTs) were finally obtained, using the negative changing rate of the HUTT as an efficacy evaluation, while in 4 of the studies, the recurrence rate of syncope or presyncope was also evaluated. A total of 233 children with VVS were included in the salt and water intervention group. The cases in the control group were treated with non-medicinal conventional therapy. The results revealed that the negative changing rate of the HUTT in the intervention group (144/233, 61.8%) was higher than that in the control group (48/179, 26.8%), and the difference was significant (*P* < 0.00001). The recurrence rate of syncope or presyncope in the intervention group (85/195, 43.6%) was lower than that in the control group (86/144, 59.7%), and the difference was significant (*P* = 0.002).

**Conclusion:** The current findings suggest that increased salt and water intake may increase the negative changing rates of the HUTT and reduce syncope or presyncope recurrence rates in pediatric patients with VVS.

## Introduction

Vasovagal syncope (VVS) is the main entity of pediatric neurally mediated syncope (NMS) and is the most common reason for pediatric syncope. Recurrent episodes of syncope can seriously affect the quality of life of children (1). As a result, the management of pediatric VVS is one of the hot topics in the research field of pediatric syncope. Health education, increased salt and water intake, pharmacological therapy, and pacemaker implantation are reported therapeutic measures for children with VVS (2), achieving therapeutic effects against one or more aspects of the supposed pathogenesis. In theory, increased salt and water intake not only directly increases plasma volume (3–5) but can also influence the regulation of cerebral and peripheral blood vessels during standing (6), which may improve tolerance to orthostatic challenge. It was also suggested that increased salt and water intake may reduce sympathetic activity during orthostatic stress, which was considered to trigger the Bezold-Jarisch reflex leading to syncope in patients with VVS (7). In addition, it is more acceptable to increase salt and water intake than to take medicines regarding the advantages of easy implementation, mild adverse reactions, and the relatively benign prognosis of VVS, especially for pediatric patients. However, the efficacy of increased salt and water intake in the treatment of pediatric VVS was inconsistent in former studies. A randomized controlled trial (RCT) in children with VVS launched by Chu et al. suggested that the head-up tilt test (HUTT)-negative changing rate was higher in the oral rehydration salts (ORS) group than in the control group (8), while Liu et al. demonstrated that there was no significant difference in the HUTT-negative changing rate between the ORS group and the control group in their study on children with VVS (9). Therefore, the aim of this study was to assess the efficacy of increased salt and water intake in the treatment of pediatric VVS and to provide more high-quality evidence for the treatment of VVS in children.

## Materials and Methods

### Inclusion Criteria

Studies were selected if the following conditions were met: (1) the diagnosis of pediatric VVS by history and positive HUTT (< 18 years); (2) RCT; (3) the patients in intervention group were treated with increased salt and water intake (including ORS, saline, and oral salt tablets) and no other medicine was prescribed; (4) the children in the control group received non-medicinal therapy without increased salt and water; (5) clinical follow-up of at least 6 months was required; and (6) the therapeutic efficacy was evaluated by the negative changing rate of HUTT and/or recurrence rate of syncope or presyncope.

### Literature Search Strategy

The databases searched by two independent coworkers (WYR and WYY) included English databases (Cochrane Library, EMBASE, PubMed, and Web of Science) and Chinese databases [VIP, Wanfang, and China National Knowledge Infrastructure (CNKI)]. The search terms (in English or Chinese) were “vasovagal syncope,” “reflex syncope,” “salt,” “sodium chloride,” “oral rehydration salts,” “hydration and salt,” “drinking water,” “saline solution,” “saline waters,” and “conventional therapy.” To obtain the literature more comprehensively, the method of combining theme words with free words was used. The retrieval date was October 5th, 2020.

### Literature Quality Evaluation

The literature quality was analyzed by the two independent reviewers mentioned above utilizing the Cochrane bias risk assessment tool. This tool was used mainly to evaluates the possible bias risks in six fields, including selection bias, implementation bias, measurement bias, follow-up bias, reporting bias, and other bias. Each study was assessed using seven established assessment indicators, including generation of random sequences, allocation concealment, blinding of participants and clinical staff, blinding of outcome assessment, incomplete outcome data, selective reporting, and other bias, each of which used “low risk,” “unclear” and “high risk” to evaluate the outcomes. A funnel plot was used to estimate the existence of publication bias. Each point on the funnel plot represents a study, the horizontal axis represents the effect size, and the vertical axis represents the standard error. A funnel plot was performed for the negative changing rate of the HUTT and the recurrence rate of syncope or presyncope.

### Data Analysis

Review Manager 5.4 software (The Cochrane Collaboration, 2020) was used for the analysis of the included studies. Chi-square test was used for heterogeneity test. If I^2^ > 50% as well as a *P*-value < 0.1, heterogeneity was identified to be meaningful, suggesting that a random effect model should be used. In contrast, if no significant heterogeneity was indicated, a fixed effect model was used. All of the included studies were RCTs, which were analyzed by the Mantel-Haenszel test to calculate the total relative risk (RR) and its 95% confidence interval (CI), with a *P*-value < 0.05 indicating a significant difference. Sensitivity analysis was applied to test the influence of each included study on the combined results and verify the stability of the strategy.

## Results

### General Information of the Included Studies

**Figure 1:**
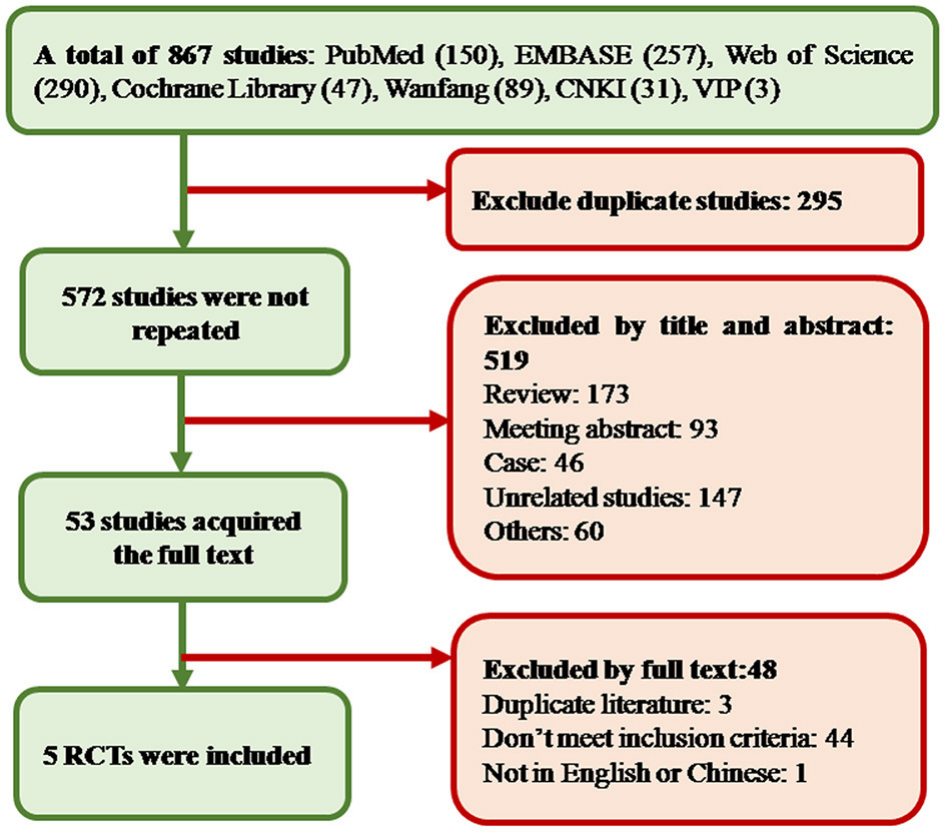
Study screening diagram. In the process of screening and selection, a total of 867 studies were obtained. Finally, five studies were included. CNKI, China National Knowledge Infrastructure; RCT, randomized controlled trial.

In total, 867 studies were obtained from 7 databases, 295 duplicates were excluded, 519 studies were excluded by screening the titles and abstracts, and the remaining 53 studies were searched in full text. Five RCTs were finally accepted, including 2 English articles and 3 Chinese articles. The flow chart of article screening is summarized in Figure 1.

Basic information of the 5 RCTs (8–12) is listed in Table 1. In total, 233 cases in the intervention group and 179 cases in the control group were included. The average patient age in all the studies ranged from 11 to 13 years old. All patients in the intervention group were treated with ORS. Each patient in the intervention group was asked to take 500 milliliters (ml) of water (standard ORS solution) orally as a supplement in addition to daily water intake. According to the components and daily dosage of ORS stated in the articles, 14.75 grams (g) of salt (containing sodium chloride 1.75 g) were supplied for each child patient per day in 3 studies (8, 9, 12), and 5.125 g of salt (containing sodium chloride 0.65 g) were supplied for each patient per day in 1 study (11). The supplementary quantity of salt was unclear in 1 study (10). The control group received non-medicinal therapy. In addition to health education and avoiding precipitating factors, children in both intervention group and control group of 3 studies (8, 11, 12) underwent upright training. All the participants in 5 RCTs were followed up for at least 6 months. The negative changing rate of HUTT (reported in 5 RCTs) and/or recurrence rate (reported in 4 RCTs) of syncope or presyncope were used as efficacy evaluations.

**Table 1:** Basic information of included studies.

**Studies**	**Study design**	**Average age (y)**	**Sex (male/female)**	**T**	**C**	**ORS**	**Water**	**Follow-up**	**Outcomes (T VS. C)**
Liu et al. 2009 (9)	RCT	11.0 ± 3.0	Unclear^#^	23	10	14.75 g/d	500 ml/d	6–12 (9.0 ± 2.1) months	N: 60.9 VS. 20.0% (*P* = 0.057) R: 52.2 VS. 60.0% (*P* = 0.722)
Meng et al. 2013 (10)	RCT	12.0 ± 4.0	Unclear^#^	30	30	Unclear	500 ml/d	6 months	N: 53.3 VS. 23.3% (*P* = 0.017) R: 36.6 VS. 50.0% (*P* = 0.297)
Chu et al. 2015 (8)	RCT	T: 12.0 ± 2.8 C: 11.8 ± 2.0	T: 35/52 C: 30/49	87	79	14.75 g/d	500 ml/d	6 months	N: 65.5 VS. 35.4% (*P* = 0.000) R: 43.7 VS. 60.8% (*P* = 0.029)
Hu et al. 2018 (11)	RCT	T: 13.0 ± 3.2 C: 12.5 ± 3.6	T: 23/32 C: 11/14	55	25	5.125 g/d	500 ml/d	2 weeks, 6 months, 12 months,	N: 70.9 VS. 24.0% (6 months) (*P* = 0.000) R: 43.6 VS. 68.0% (12 months) (*P* = 0.043)
Li et al. 2019 (12)	RCT	11.9 ± 2.8	32/41	38	35	14.75 g/d^*^	500 ml/d^*^	6–25 (14.8 ± 6.1) months	N: 47.4 VS. 14.3% (*P* = 0.002)

*N represents the negative changing rate of the head-up tilt test, R represents the recurrence rate of syncope or presyncope, T represents the treatment group, and C represents the control group*.

# *The specific sex ratio was not available, but Liu 's study noted that there was no significant difference between the two groups*.

* *A half dose was prescribed for children < 6 years old*.

*RCT, randomized controlled trial; ORS, oral rehydration salts*.

### Quality Evaluation of the Included Studies

**Figure 2:**
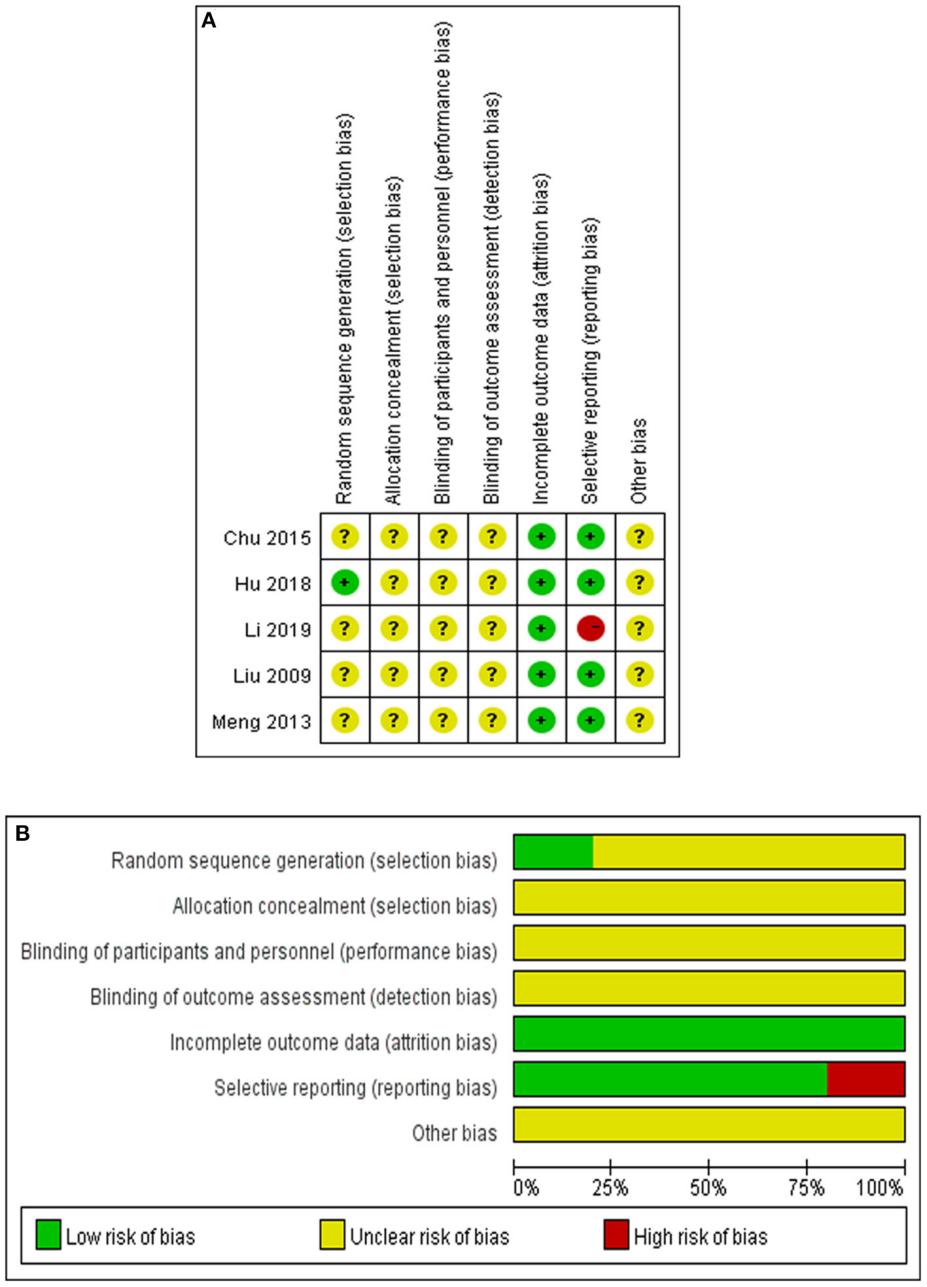
Quality evaluation of the studies. **(A)** Summary of bias risk in each study. Evaluation of the bias risk was based on the reviewers' assessments of seven items for each selected study as follows. “+” implies the existence of a low risk, “-” implies the existence of a high risk, “?” implies unclear risk bias because of a lack of relevant information. **(B)** Summary of bias risk in each item. Evaluation of the bias risk for each item was based on reviewers' assessments of five studies for each bias risk item and are presented as percentages.

A total of 5 RCTs were evaluated for quality analysis. Only in one study (11) the method of random sequence generation was described, and in other studies the process of random allocation was not described. Allocation concealment and blindness were not mentioned in any of the 5 studies. All of studies reported the loss ratio of follow-up. Four studies (8–11) were assessed as low risk because there was no selective reporting, and one study (12) was assessed as high risk because of incomplete reporting of outcomes. It was not clear whether there were other biases among the included studies (Figure 2A). In addition, risk bias was summarized and expressed as a percentage (Figure 2B).

### Publication Bias Evaluation

**Figure 3:**
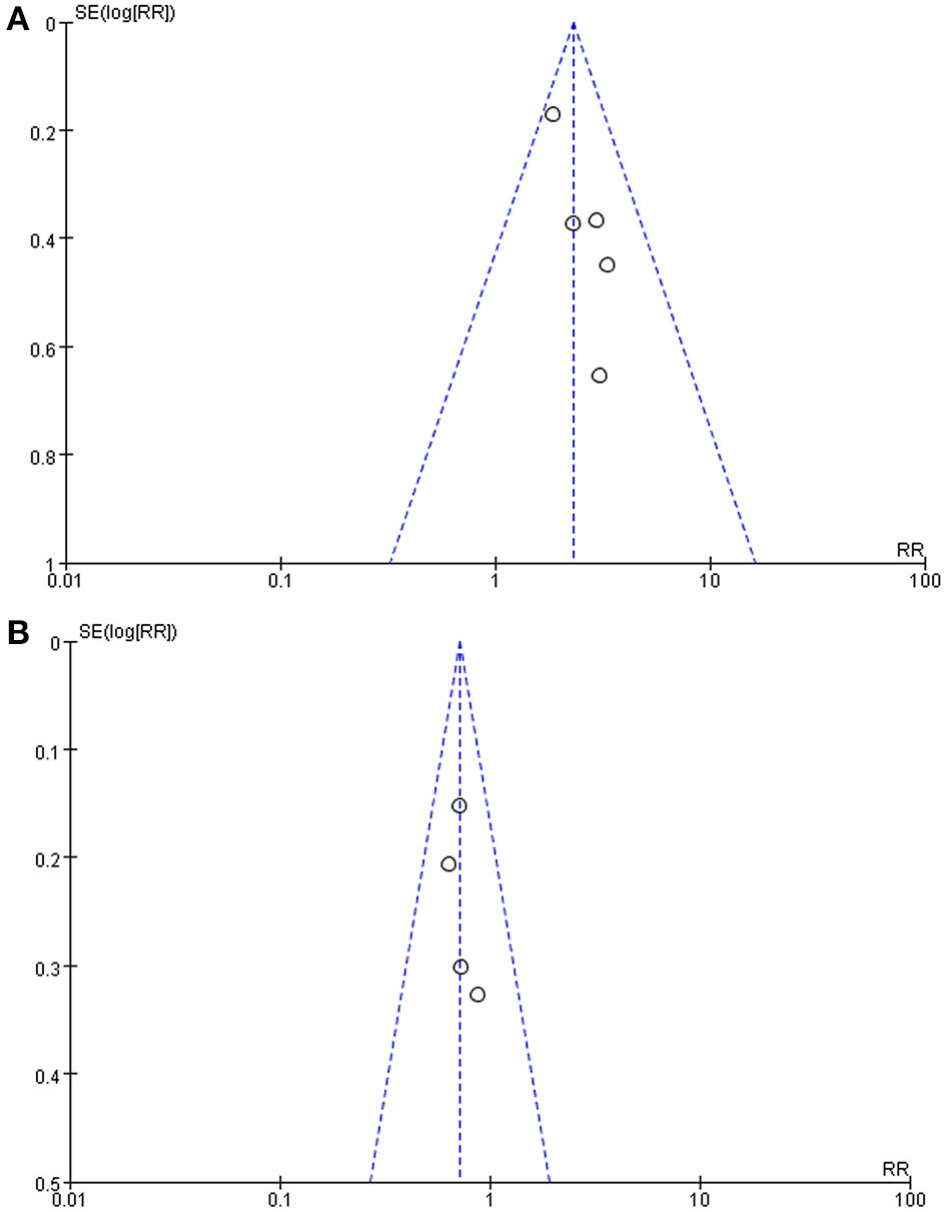
Funnel plot of the studies. **(A)** Funnel plot of studies using the negative changing rate of the HUTT as the efficacy evaluation. **(B)** Funnel plot of studies taking syncope or presyncope recurrence rate as the efficacy evaluation. Each dot on the funnel plot represents a study, the horizontal axis represents the effect size, and the vertical axis represents the standard error. As shown in the plot, each study was close to the ideal effect value on the horizontal axis as they were evenly distributed on both sides, and each study had a low standard error on the vertical axis. SE, standard error; RR, relative risk.

Publication bias was evaluated by funnel plots (Figure 3). It can be seen from the figures that there was no significant publication bias either in studies evaluating the response of the HUTT or in studies following up syncope or presyncope recurrence of the patients.

### Efficacy Evaluation

**Figure 4:**
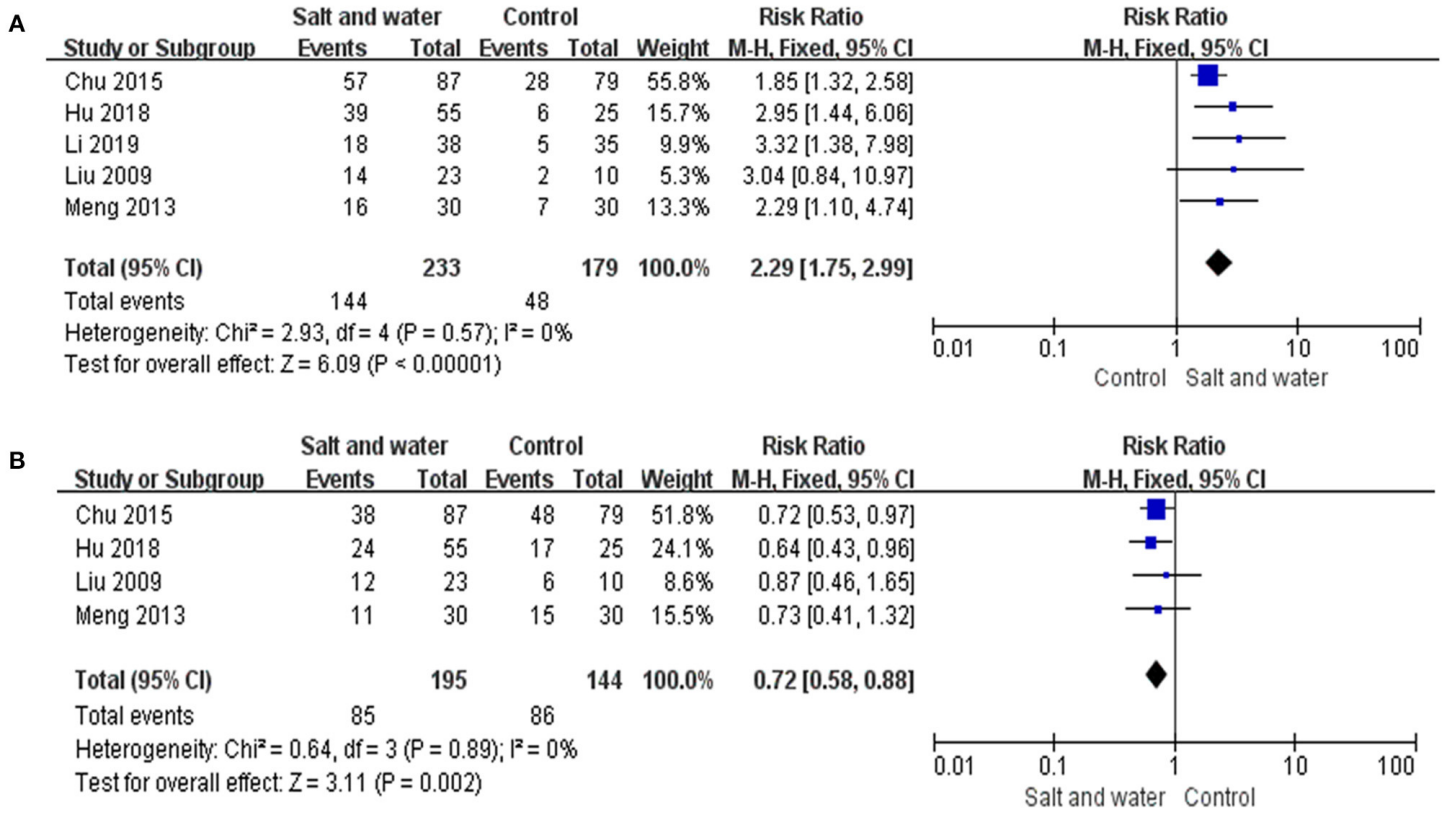
Forest plot of the studies. **(A)** Forest plot of the studies evaluating therapeutic efficacy of increased salt and water intake with the negative changing rate of HUTT. **(B)** Forest plot of the studies evaluating the therapeutic efficacy of increased salt and water intake with syncope or presyncope recurrence rate. Heterogeneity analysis showed no conspicuous heterogeneity among the above studies, and a fixed-effect model was adopted. The RR results analyzed by the M-H test are summarized on the right. The RR and its 95% CI of each study are represented by small squares and transverse lines, respectively, and the rhombus below represents the overall results of the meta-analysis. RR, risk ratio; CI, confidence interval; M-H, Mantel-Haenszel test.

Five studies used the negative changing rate of the HUTT, and 4 studies used the recurrence rate of syncope or presyncope as an evaluation of therapeutic effects. Taking response in the HUTT as a therapeutic outcome, a total of 412 children were analyzed, including 233 in the intervention group and 179 in the control group, among which 144 and 48 turned negative response in the HUTT after treatment, respectively. The heterogeneity test showed no significant heterogeneity among the included studies (Chi^2^ = 2.93, *P* = 0.57, I^2^ = 0%). Therefore, a fixed effect model as well as RR values were selected for outcome analysis. The evidence of the meta-analysis revealed that the HUTT negative changing rate in the intervention group was remarkably higher than that in the control group (RR: 2.29, 95% CI: 1.75–2.99, *P* < 0.00001) (Figure 4A). A total of 339 children evaluated by syncope or presyncope recurrence rate were analyzed, including 195 in the intervention group and 144 in the control group, among which 85 and 86 reported recurrence during follow-up, respectively. The heterogeneity test showed no obvious heterogeneity among the included studies (Chi^2^ = 0.64, *P* = 0.89, I^2^ = 0%). The evidence of the meta-analysis revealed that the syncope or presyncope recurrence rate in the intervention group was remarkably lower than that in the control group (RR: 0.72, 95% CI: 0.58–0.88, *P* = 0.002) (Figure 4B). Sensitivity analysis was done by re-analyzing the data when excluding each single study one by one. The results showed that, no matter which single study is excluded, the combined effects on the recurrence rate of syncope or presyncope and the HUTT negative changing rate of the remaining studies were the same as that of all the 5 RCTs, indicating no significant heterogeneity.

**Figure 5:**
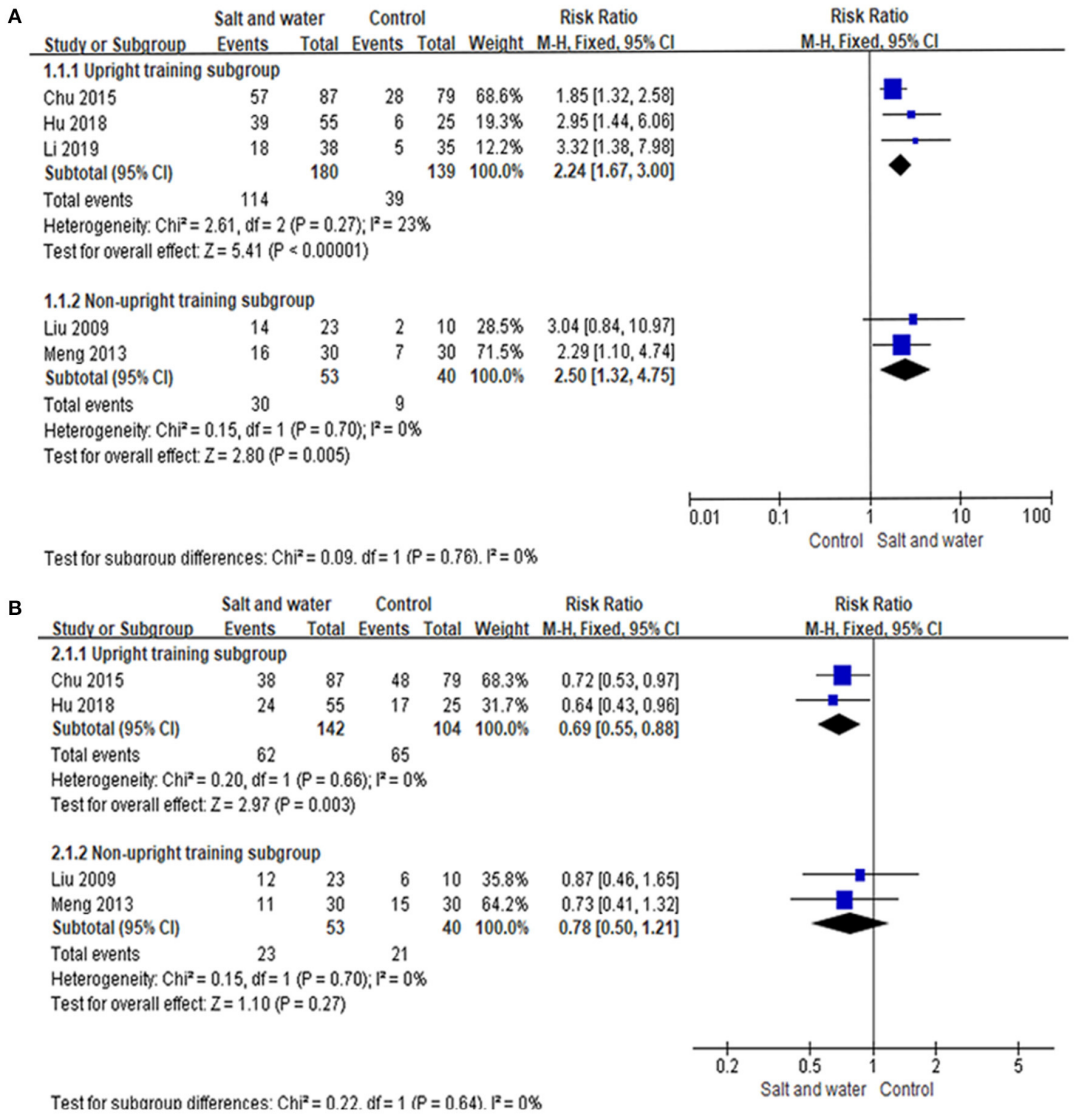
Forest plot of the subgroup analysis. **(A)** Forest plot of the studies evaluating the therapeutic efficacy of different subgroup with the negative changing rate of HUTT. **(B)** Forest plot of the studies evaluating the therapeutic efficacy of different subgroup with the syncope or presyncope recurrence rate. Heterogeneity analysis showed no conspicuous heterogeneity among the above studies, and a fixed-effect model was adopted. The RR results analyzed by the M-H test are summarized on the right. The RR and its 95% CI of each study are represented by small squares and transverse lines, respectively, and the rhombus below represents the overall results of the meta-analysis. RR, risk ratio; CI, confidence interval; M-H, Mantel-Haenszel test.

As the participants in 3 studies received upright training, the studies were divided into two subgroups according to whether upright training was suggested. The meta-analysis on the 2 studies without upright training showed that the negative changing rate of the HUTT in the intervention group was higher than that in the control group (RR: 2.50, 95% CI: 1.32–4.75), and the difference was significant (*P* = 0.005). The recurrence rate of syncope or presyncope did not differ between the two groups (RR: 0.78, 95% CI: 0.50–1.21, *P* = 0.27), and there was no heterogeneity between the two studies (Chi^2^ = 0.15, *P* = 0.70, I^2^ = 0%). The analysis on the other 3 studies using upright training in the participants revealed that the negative changing rate of the HUTT in the intervention group was higher than that in control group (RR: 2.24, 95% CI: 1.67–3.00, *P* = 0.000), and the recurrence rate of syncope or presyncope in the intervention group was lower than that in the control group (RR: 0.69, 95% CI: 0.55–0.88, *P* = 0.003). There was no heterogeneity among the three studies (Chi^2^ = 2.61, *P* = 0.27, I^2^ = 23%) (Figure 5).

### Adverse Reactions and Compliance

Three studies (8, 9, 12) mentioned adverse reactions in children treated with increased salt and water intake, and no relevant adverse reactions were reported. Two studies (9, 12) described the compliance of the patients, revealing that all the pediatric patients in the intervention group showed good compliance.

## Discussion

Increased salt and water intake was recommended as one of the primary therapeutic measures for children with VVS. According to the pathophysiology of VVS, decreased venous return to the heart during orthostatic positioning, which can be aggravated by relatively insufficient blood volume, will lead to abnormal excitement of the cardiac vagal nerve and the occurrence of syncope (13). Thus, it is theoretically believed that salt and water supplementation can increase the blood volume and achieve certain therapeutic efficacy in children with VVS. However, as mentioned above, inconsistent results were reported regarding the efficacy of increased salt and water intake. A meta-analysis published in 2020 (14) evaluated the efficacy and safety of salt intake in the treatment of patients with orthostatic intolerance aged 11–65 years, suggesting that although the safety was excellent, the efficacy was poor. However, the results of this analysis can hardly be applied to children with VVS because the dominant research population was adults, and the etiological heterogeneity of orthostatic intolerance should be taken into consideration. In this study, five RCTs were selected for meta-analysis, and the evidence showed that the efficacy of increased salt and water intake was superior to that of the control group, which could both significantly improve the response in the HUTT and reduce the recurrence of syncope or presyncope. The above results all suggested that salt and water intake was an effective measure in the management of children with VVS.

Despite the better efficacy of salt and water intake compared with conventional therapy in the treatment of pediatric VVS, the negative changing rate of HUTT and symptom-free rate were not as high, 61 and 56%, respectively, in summary of the 5 RCTs. Several aspects, such as the baseline symptom severity, the amount of salt and water supplement, the complex influence of daily diet, the different hemodynamic type of VVS, and patient compliance, may affect the final outcomes of ORS therapy and should be considered during the research design. As showed in the results, without upright training in both groups, children in the intervention group showed no difference in the recurrence rate of syncope or presyncope compared with the controls although the negative changing rate of the HUTT was still high. However, with upright training in both groups, the recurrence rate of syncope or presyncope was lower in the intervention group than that in the control group. More researches are needed to explore whether better therapeutic effects can be achieved by the combined therapy of ORS and upright training. We also found that ORS therapy had been compared with other medicine therapies in some previous studies. For example, Kaufmann and Zhang both showed that the effective rate of midodrine could reach 70–80% (15, 16), and another RCT study suggested that the effective rate of metoprolol in the treatment of pediatric VVS was 89.29% (17). Although midodrine or metoprolol seemed to have better efficacy compared with increased salt and water intake in these studies (18, 19), the latter still has its irreplaceable advantages. It can be easily accepted by children and parents and has many fewer side effects than medicines, resulting in better compliance and safety, especially in long-term use. In addition, it is believed that increased salt and water intake alone may achieve a better effect on pediatric patients who are deemed to have relatively lower blood volume, while children with other predominant pathogeneses, such as excessive vasodilation and autonomic nervous dysfunction, may respond better to corresponding medicines (13). Furthermore, the influence of hemodynamic types of pediatric VVS according to the response during HUTT was also considered in three of the five included studies. In Li's study, the efficacy of ORS in children with vasodepressor type of VVS was superior to those with mixed or cardioinhibitory type of VVS (12). However, Hu's study showed that there was no statistical difference among children with different hemodynamic types of VVS (11), which was also consistent with Chu's research (8). Considering these conflicting results, we can't draw a unified conclusion and need further researches to investigate the efficacy of ORS in treating children with different hemodynamic types of pediatric VVS. Therefore, a comprehensive assessment should be carried out before the treatment of VVS in children. As a result, for children with relatively mild symptoms who first visit clinics, increased salt and water intake can be considered the primary therapy, while for children with refractory VVS, individualized treatment based on its pathogenesis may be a more reasonable choice.

Currently, there is no uniform recommendation on the amount of salt and water intake for children with VVS. Therefore, in our analysis, we took special attention to this issue. The 2018 European Society of Cardiology (ESC) Guideline on syncope management recommended 2–3 liters of fluid and 10 g of sodium chloride daily for adults with orthostatic hypotension and orthostatic intolerance (20). According to the latest Chinese dietary guidelines published in 2016 (21), 600–800 ml water and 800–1,400 ml water were recommended for preschool children and school children per day, respectively, except for milk and other drinks. Our analysis summarized the supplementary amount of water in treating children with VVS and showed that an extra intake of 500 ml water in addition to daily requirements can be beneficial. The amount of salt intake needs more deliberate consideration. A maximum amount of 5.8 g of sodium chloride was recommended for children and adolescents by the American Academy of Pediatrics in 2017 (22). However, some children with VVS are presumed to have insufficient daily salt intake. Therefore, an added supplement of salt is necessary. In our analysis, the supplementary amount of salt was 5.125 g−14.75 g, containing 0.65 g−1.75 g of sodium chloride. We further compared the negative changing rate of the HUTT and recurrence rate of syncope or presyncope between the participants who were supplemented with 5.125 g and 14.75 g of salt per day in the included studies, and no significant difference was found (negative changing rate of the HUTT: Chi^2^ = 1.998, *P* = 0.158; recurrence rate of syncope or presyncope: Chi^2^ = 0.049, *P* = 0.825). It seemed that the supplementary amount of salt didn't influence the results. However, expanded sample size is needed to confirm these results because participants in only one study were supplemented with 5.125 g of salt per day. Meanwhile, it is difficult to accurately estimate the total salt intake for children in clinical studies, so certain biomarkers of sodium load in the body, such as 24-h urine sodium levels, can be investigated to select patients who may respond well to salt supplementation (23).

In addition, we also paid attention to the side effects of ORS therapy. It has been reported that salt intake at a high dose (taking 154 g of salt in 4 days) can cause hypernatremia (24), and it is known that long-term high-salt intake may increase the risk of hypertension. However, no significant adverse reactions were found in the included studies at the mentioned dose during the 6-month follow-up. Therefore, it may be necessary to monitor the 24-h urinary sodium and blood pressure of children regularly during salt supplement therapy.

As mentioned above, ORS I or ORS III was used in the included studies. One study compared the efficacy of ORS I and ORS III, but there was no significant difference between the two types of ORS in the therapeutic outcome (25). Delving into ORS components, in addition to sodium ions, other ions, such as potassium ions, chloride ions, and glucose, were included in our included studies. Potassium ions might also play a role in the treatment of children with NMS. A randomized double-blind placebo-controlled study suggested that both potassium chloride and potassium bicarbonate could improve the vascular endothelial function of patients and have beneficial effects on the cardiovascular system (26). Nevertheless, it is not clear at present whether other ions or glucose have effects on patients.

In terms of literature quality and study design, no heterogeneity or publication bias was found among the 5 RCTs, and only in 1 study the randomized method was described well. None of the 5 studies conducted blind methods, which can lead to some degree of bias. However, the sensitivity analysis indicated that our results were consistent and reliable.

In addition, this study also has some limitations. First, non-Chinese and non-English studies, and the unpublished studies were not included, which may have led to incomplete databases. Second, positive results are more likely to be published, which may lead to publication bias and affect the final results. Finally, in these 5 open-label studies, some baseline characteristics of the participants, such as ethnicity, daily intake of salt and water, hemodynamic type of pediatric VVS and comorbidities, were not fully analyzed because of the limited data, which might have some influence on the outcomes.

## Summary

In summary, increased salt and water intake can significantly reduce the recurrence rate of syncope or presyncope and increase the negative changing rate of HUTT, and it is suggested to be the first-line treatment for pediatric VVS. Although effective, 1/3–1/2 of patients still have poor therapeutic outcomes. In addition, the treatment of increased salt and water intake is safe and had good patient compliance in our analysis, however, possible side effects, such as hypernatremia and hypertension, should be monitored. More well-designed studies are needed not only to evaluate the therapeutic efficacy of increased salt and water intake in treating children with VVS but also to explore the proper amount and duration of salt and water supplementation according to individual baseline status to produce a more efficient treatment strategy for children with VVS.

## Data Availability Statement

The original contributions presented in the study are included in the article/supplementary material, and further inquiries can be directed to the corresponding authors.

## Author Contributions

YL, HJ, and HZ conceived the idea and revised and reviewed the manuscript. JD, HZ, YL, and HJ supervised the program. YaW and YuW wrote the meta-analysis, searched the literatures, and evaluated their quality. XL gave guidance on statistics. All authors contributed to the article and approved the submitted version.

## Conflict of Interest

The authors declare that the research was conducted in the absence of any commercial or financial relationships that could be construed as a potential conflict of interest.
